# Whole Mitochondrial Genome Analysis in Serbian Cases of Leber’s Hereditary Optic Neuropathy

**DOI:** 10.3390/genes11091037

**Published:** 2020-09-02

**Authors:** Phepy G. A. Dawod, Jasna Jancic, Ana Marjanovic, Marija Brankovic, Milena Jankovic, Janko Samardzic, Dario Potkonjak, Vesna Djuric, Sarlota Mesaros, Ivana Novakovic, Fayda I. Abdel Motaleb, Vladimir S. Kostic, Dejan Nikolic

**Affiliations:** 1 Faculty of Medicine, University of Belgrade, 11000 Belgrade, Serbia; dr.phepy@gmail.com (P.G.A.D.); jasna.jancic.npk@gmail.com (J.J.); ana.marjanovic@yahoo.com (A.M.); mara.brankovic@gmail.com (M.B.); dariopotkonjak10@gmail.com (D.P.); sharlotam@gmail.com (S.M.); ivana.novakovic@med.bg.ac.rs (I.N.); vladimir.s.kostic@gmail.com (V.S.K.); 2 Department of Medical Biochemistry and Molecular Biology, Faculty of Medicine, Ain Shams University, Cairo 11591, Egypt; dr.fayda@hotmail.com; 3 Clinic of Neurology and Psychiatry of Children and Youth, 11000 Belgrade, Serbia; vesna.djuric84@gmail.com; 4 Neurology Clinic, Clinical Center of Serbia, 11000 Belgrade, Serbia; milena.jankovic.82@gmail.com; 5 Institute of Pharmacology, Clinical Pharmacology and Toxicology, Faculty of Medicine, University of Belgrade, 11000 Belgrade, Serbia; jankomedico@yahoo.es; 6 Physical Medicine and Rehabilitation Department, University Children Hospital, Tirsova 10, 11000 Belgrade, Serbia

**Keywords:** Leber’s hereditary optic neuropathy, mtDNA, mutations, haplogroups

## Abstract

Leber’s hereditary optic neuropathy (LHON) is a maternally inherited disorder that affects central vision in young adults and is typically associated with mitochondrial DNA (mtDNA) mutations. This study is based on a mutational screening of entire mtDNA in eight Serbian probands clinically and genetically diagnosed with LHON and four of their family members, who are asymptomatic mutation carriers. All obtained sequence variants were compared to human mtDNA databases, and their potential pathogenic characteristics were assessed by bioinformatics tools. Mitochondrial haplogroup analysis was performed by MITOMASTER. Our study revealed two well-known primary LHON mutations, m.11778G>A and m.3460G>A, and one rare LHON mutation, m.8836A>G. Various secondary mutations were detected in association with the primary mutations. MITOMASTER analysis showed that the two well-known primary mutations belong to the R haplogroup, while the rare LHON m.8836A>G was detected within the N1b haplogroup. Our results support the need for further studies of genetic background and its role in the penetrance and severity of LHON.

## 1. Introduction

Leber’s hereditary optic neuropathy (LHON; OMIM 535000), is a neurodegenerative inherited form of painless loss of central vision in both eyes, which may happen simultaneously or sequentially [1]. LHON was first revealed in 1871 by a German ophthalmologist, Theodor Leber, who first described the maternal transmission of the disorder [2]. Now, it is explained as a non-Mendelian genetic pattern of mitochondrial inheritance [3,4,5]. Mitochondria have their own DNA (mtDNA) that encode several subunits of large enzyme complexes in the mitochondria oxidative phosphorylation pathway system (OXPHOS), which takes place in the inner membrane of mitochondria that catalyze ATP synthesis and provide cellular energy [6,7]. Missense mutations in mtDNA genes associated with LHON cause mitochondrial dysfunction with drastic defects in complex I, the largest complex in the OXPHOS [8], leading to increased reactive oxygen species (ROS) and a marked reduction in the ATP supply of the retina. Injury of retinal neurons [9,10,11] and degeneration of the optic nerve lead to the specific features of LHON [12]. 

LHON point mutations in complex I genes such as m.3460G>A, m.11778G>A, and m.14484T>C in *MT-ND1*, *MT-ND4*, and *MT-ND6* genes, respectively [13], are considered the most prevalent primary LHON mtDNA mutations, as they account for 90% of LHON patients [14,15]. They have variable prognoses for the spontaneous recovery of visual loss [16] withy m.11778G>A having the worst overall visual prognosis [17]. Moreover, there are other mtDNA mutations elsewhere in the mitochondrial genome such as m.3635G>A, m.4171C>A, m.8836A>G, m.10663T>C, m.14502T>C, and m.14597A>G that are considered as rare causes of LHON within the population [18,19,20,21,22,23]. Unlike other pathogenic mtDNA mutations, primary LHON-mtDNA mutations occur nearly or completely homoplasmically [24,25]. Furthermore, these primary LHON mutations predominantly show incomplete penetrance, which results in inter-individual variability in the age at onset, severity and progression of visual impairment for the same LHON-mtDNA mutation, even for matrilineal relatives [26,27].

There has been growing interest in the co-occurrence of incomplete penetrance of the primary LHON mutations and secondary LHON-associated mtDNA mutations. Secondary mutations have been studied as modifiers for phenotypic expression of LHON by acting in synergy with the primary mutations. In addition, intermediate LHON mutations might act independently of the occurrence of primary mutations [28,29,30]. Other mitochondrial variants such as m.3394T>C, m.4435A>G, m.5601C>T and m.15951A>G have been found to be strongly associated with positive 11778G>A LHON disease [31,32,33,34]. In addition, the specific mtDNA haplogroup of a number of successive secondary mutations has been analyzed in order to detect their possible association with particular primary mutations and to analyze their influence on mutation penetrance and disease expression. In the European background, haplogroup J has been shown to be a penetrance enhancer and to be highly susceptible to the pathogenic effects of *ND4/*11778 and *ND6/*14484 LHON primary mutations; in contrast, the *ND1/*3460 LHON mutation has a slight tendency to haplogroup J [35].

Previously, the first population-based study on LHON in the Serbian population were done by Jancic et al. [36,37] who shed light on the frequencies of primary LHON mutations in the population of Serbia, and confirmed the predominance of males among affected patients. Here we present the results of further molecular-genetic study, which asserts the significance of both primary and secondary mutations in the mtDNA genome in association with mitochondrial haplotypes for better characterizing the clinical manifestations and molecular features of LOHN-associated phenotypes.

## 2. Materials and Methods

### 2.1. Study Population

**Figure 1:**
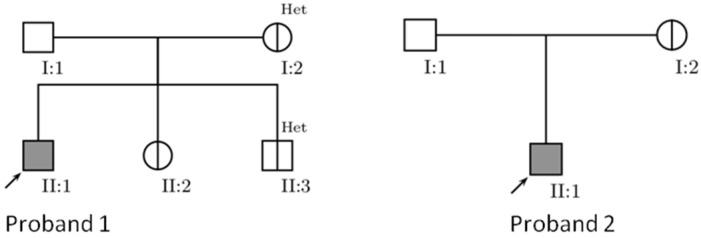
Pedigrees of two analyzed Leber’s hereditary optic neuropathy (LHON) families. I:1 not assessed, a halved circle/square = asymptomatic mutation carrier, Het = heteroplasmy.

This study included a total of eight Serbian probands (marked as P1–P8) clinically affected and genetically confirmed with LHON and four asymptomatic LHON mutation carriers (relatives of probands P1 and P2) (Figure 1). Respondents were recruited in the period from February 2013 to February 2019 from the Clinic for Neurology and Psychiatry for Children and Youth and the Clinic of Neurology, Clinical Center of Serbia (CCS), Belgrade, Serbia. Comprehensive neurological and ophthalmologic examinations were performed in all respondents, including specialized visual testing, fundus examination for the optic disc and vascular changes, and brain imaging. Demographic data, habits and risk factors such as smoking and alcohol were evaluated as well as past history for head trauma and additional systematic illnesses or drug consumption. A clear family history of visual loss affecting maternal relatives was noted. All molecular genetic investigations were performed in the Laboratory for Genetic and Molecular Diagnostics of Neurological Disorders, Neurology Clinic CCS, Belgrade. Convenient informed consent was obtained from all of the participants and this was reviewed by the Ethical Committee (Number: 2650/VI-1) of the Faculty of Medicine, University of Belgrade before ethical approval was given for this study.

### 2.2. DNA Extraction

Peripheral blood (5 mL) was collected from each respondent. DNA was extracted from a 200 μL blood sample following the manufacturer’s protocols (Invitrogen, Thermo Fisher Scientific, Waltham, MA, USA, https://www.thermofisher.com). In the first step, 20 μL of proteinase K and 20 μL of RNAase was added, and the mixture was briefly vortexed and centrifuged. Then, 200 μL of PureLink™ Genomic Lysis/Binding Buffer was added and the sample was incubated for 10 min at 56 °C. After that, 200 μL of 99% ethanol was added, the sample was briefly vortexed and centrifuged, and transferred to the PureLink™ Genomic spin column. After being centrifuged for 1 min at 9700 rpm, the collection tube was discarded and the spin column was transferred into a clean PureLink™ Collection Tube supplied with the kit. Ethanol 96–100% was added to PureLink™ Genomic Wash Buffer 1 and Buffer 2 according to the instructions on each label. Then, 500 μL Wash Buffer 1 was added to the column, centrifuged for 1 min at 9700 rpm, after which the column was transferred to a collection tube of 2 mL; 500 μL Wash Buffer 2 was added to the column, centrifuged for 3 min at 13,000 rpm, and the spin column was transferred to a sterile 1.5 mL micro centrifuge tube. Finally, 100 μL of deionized water was added to the column, incubated for 5 min at room temperature, then centrifuged for 2 min at 13,000 rpm, removed and the column was discarded. The purified genomic DNA sample was stored at 4 °C for immediate use or at −20 °C for long-term storage.

### 2.3. DNA Amplification by Polymerase Chain Reaction

The whole human mitochondrial genome (np 1–16569) was amplified by polymerase chain reaction (PCR), and 28 pairs of M13-tagged oligonucleotide primers were used to produce entire overlapping segments that were each in the range of 600 to 700 bp [38]. PCR was performed by adding 1 µL of patient DNA sample to a total volume of 12.5 µL solution containing 10× DreamTaq Buffer; 0.2 mM deoxynucleoside triphosphates (dNTPs), 0.5mM of appropriated both forward and reverse primers, 0.5 U of DreamTaq DNA Polymerase (Thermo Fisher Scientific, Waltham, MA, USA) and 15 µg of bovine serum albumin (BSA). We noticed that this PCR regimen effectively worked with all 28 PCR amplified fragments except segment number 23, which worked with a special, optimized PCR program that preferentially works with Taq™ DNA polymerase. We ran the touchdown PCR amplification protocol. The program consisted of an initial cycle of denaturation for 5 min at 95 °C, then 5 cycles of hybridization for 30 s at 63 °C, 30 cycles of 30 s at annealing temperatures that decreased gradually from 63 °C to 57 °C, followed by 5 cycles for 30 s at 57 °C and then a holding period at 4 °C. Gel electrophoresis was done to reconfirm the targeted extended PCR products followed by ExoSAP for the enzymatic purification of amplicons as a preparatory step for fluorescence-based cycle sequencing based on the Sanger methodology. 

Fluorescence-based cycle sequencing was performed by applying BigDye Terminator v3.1 Cycle Sequencing Kit to produce the amplified extension products that are terminated by one of the four dideoxynucleotides (ddNTPs), followed by alcohol-based nucleic acid ethanol precipitation for more purification of the amplified products according to the standard protocols of Applied Biosystems.

### 2.4. Capillary Electrophoresis

We used capillary electrophoresis on the ABI Prism 3500 Genetic Analyzer instrument (Applied Biosystems, Waltham, MA, USA) for automated DNA sequencing. All fragments were sequenced in forward and reverse directions by using their specific primers at least twice for confirmation of a detected variant.

### 2.5. Bioinformatics Analysis

After alignment, the mtDNA variants were compared with the Revised Cambridge Reference Sequence (rCRS), (NCBI Reference Sequence: NC_012920) [39] by Sequencher DNA Sequence Analysis Software, and subsequently, the mitochondrial variants were analyzed by the MITOMAP database system for the human mitochondrial genome (http://www.mitomap.org/MITOMAP) [40]. The Human Mitochondrial Genome Database (mtDB) is another resource for human mitochondrial variants (http://www.genpat.uu.se/mtDB) [41]. Moreover, the GenBank for Human Mitochondrial Genome Database (http://www.ncbi.nlm.nih.gov/Genbank/index.html) [42] and MEDLINE-listed publications on life sciences can also be used for identified variants. 

The pathogenic characteristics of the primary mutations and non-synonymous polymorphic mtDNA variants were assessed by protein-based metrics in silico predictive software. Human amino acid reference sequences were identified in the Universal Protein Resource (UniProt) (http://www.UniProt.org) [43]. Three different prediction tools available online were applied: PolyPhen2, PANTHER and PROVEAN. The polymorphism PolyPhen-2 database for reported mtDNA mutations (Polymorphism Phenotyping v2, http://genetics.bwh.harvard.edu/pph2/) was used for predicting the implicated drastic effects of missense mutations on the structure and function of human proteins (HumVar) [44]. Protein Analysis Through Evolutionary Relationships (PANTHER), (http://pantherdb.org/panther/summaryStats.jsp) was used as a source for evolutionary history classification of the protein sequences [45]. The Protein Variation Effect Analyzer (PROVEAN) (http://provean.jcvi.org) server was used for predicting the impact of amino acid substitutions. Variants with scores less than −2.5 were considered deleterious [46].

### 2.6. Haplogroup Analysis

Haplogroup analysis was done for all included subjects. FASTA-formatted files were submitted to MITOMASTER to identify nucleotide variants relative to the rCRS for determination of the predicted haplogroups (https://www.mitomap.org/foswiki/bin/view/MITOMASTER/WebHome) [47]. All the sequence variations in the studied probands were also presented in a tree profile PhyloTree build 17 (https://www.phylotree.org/) was used as this is a widely accepted, comprehensive phylogenetic tree of global human mitochondrial DNA that is suitable for alignment and haplogroup estimation [48].

## 3. Results

### 3.1. Determination of Primary and Secondary LHON Pathogenic Mutations

This study provided mtDNA screening for pathogenic mutations and variants associated with LHON in a total of 12 Serbian subjects; six of them are grouped in two pedigrees (included two probands, P1 and P2, and four mutational carriers, see Figure 1) and six singleton probands (P3–P8). Our analysis revealed two of the most frequent primary mutations shared in LHON, m.11778G>A in *MT-ND4* and m.3460G>A in *MT-ND1*, as well as one mutation m.8836A>G in *MT-ATP6* which is consider to be a rare cause of LHON. Change m.11778G>A was detected in both observed families and in four individual probands, while two other mutations were each observed in a single affected case, with a total frequency of 83.33%, 8.33%, and 8.33% respectively. Among the affected subjects, seven are males and one female, the latter with m.3460G>A primary change. All probands showed homoplasmic primary mutations. The homoplasmic to heteroplasmic ratio in the entire study group was 10:2; heteroplasmy was revealed in two asymptomatic carriers of the m.11778G>A mutation (Figure 1).

Besides the detection of primary mutations, complete mtDNA analysis by Sanger sequencing was done. Our analysis revealed five secondary nonsynonymous mutations: m.3394T>C in *ND1*, m.4216T>C in *ND1*, m.13708G>A in *ND5*, m.15257G>A in *MT-CYB*, and m.15812G>A in *MT-CYB*. Changes at nucleotide positions 4216 and 13708 were linked with both m.3460G>A and m.11778G>A primary mutations, while m.3394T>C (Y30H) was linked with m.11778G>A only; other changes were individually linked with the same primary mutations (Table 1). Four associated mutations for other diseases were also detected. The m.988G>A and m.15287T>C are a deaf risk factor and helper mutation, respectively; m.2755A>G is possibly associated with left ventricular non-compaction cardiomyopathy (LVNC), and m.3796A>G is connected to adult-onset dystonia. There was no specific pattern of secondary or associated mutations in affected vs. unaffected subjects. Among other risk factors, moderate alcohol and cigarette consumption were noticed in two probands (P4 and P7). 

### 3.2. Bioinformatics Analysis for LHON Associated Mutations

The bioinformatics analysis of all primary/rare LHON pathogenic mutations and the evaluation of downstream secondary LHON mutations was done by using in silico predictive software. Human amino acid reference sequences were identified in UniProt as follows: P03886, P00846, P03905, P03915, and P00156. Based on the PolyPhen2 assessment score, LHON pathogenic mutations m.3460G>A and m.11778G>A were classified as definitely pathogenic according to the HumVar model while m.8836A>G was classified as possibly damaging, with scores of 1.000, 0.996, and 0.770, respectively. The other changes were recorded as neutral or benign variants. Additional analysis of those secondary mutations that are implicated in the disease by changing highly evolutionary conversed amino acid was done by using PANTHER, which confirmed the generality of the relationship between the secondary mutations and the changes in the corresponding conversed amino acids; however, PROVEAN determined that the m.3394T>C, m.11778G>A, and m.15257G>A variants were deleterious (Table 2).

### 3.3. MITOMASTER Analysis for Associated Mitochondrial Haplotypes

**Figure 2:**
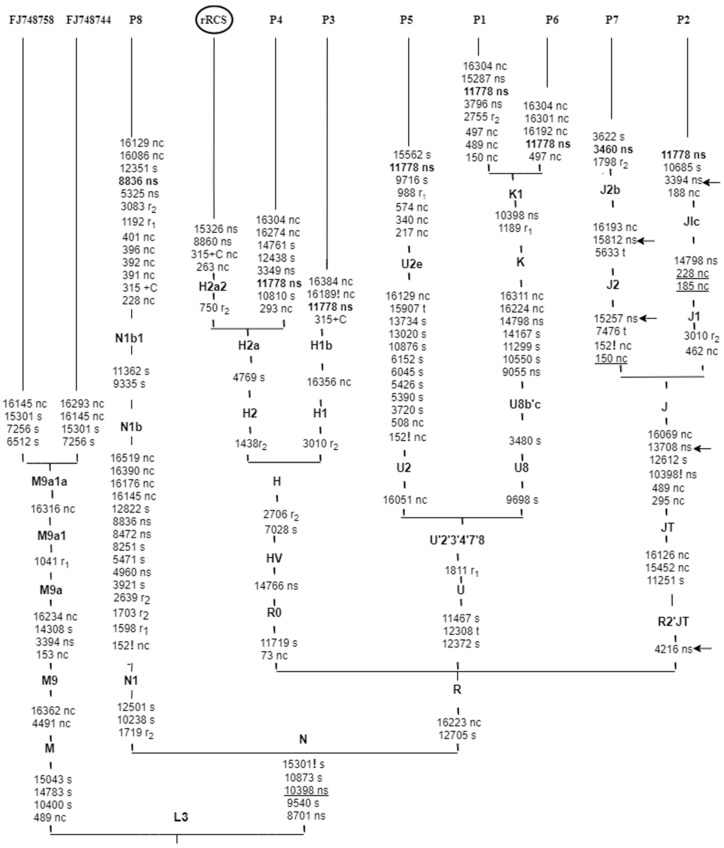
Schematic phylogenetic tree encompassing the complete mtDNA genome from eight Serbian probands with pathogenic LHON mutations. The tree was rooted by following the nomenclature of mtDNA tree Build 17, using the reference sequence rCRS haplogroup (H2a2a). Underlining indicates recurrent mutations; suffixes indicate back mutations (!), non-coding region (nc), synonymous variants (s), nonsynonymous variants (ns). Variations in the transfer RNA and the ribosomal RNA1, and 2 genes are denoted by (t), (r_1_), and (r_2_), respectively. Previous mtDNA sequences that have showed the association of m.3394 variant on haplogroup M9a background in Chinese families, are added (GenBank accession number FJ748744 and FJ748758, [49]). The arrows indicate the nonsynonymous mutations that might have a potential role in LHON expression. LHON primary mutations are shown in bold and were added to the tree after phylogeny tree construction.

In our study, the most frequent LHON primary mutation m.11778 was associated with different haplogroups belonging to groups H, U and J, all branches of the mitochondrial macro-haplogroup R. The single case of mutation m.3460 was associated with haplogroup J2, part of group J, subgroup JT. Mutation m.8836 was associated with haplogroup N1b, a branch of macro-haplogroup N (Table 3, Figure 2). 

### 3.4. Phenotypic Characterization in Symptomatic and Asymptomatic Subjects

A comprehensive clinical examination was performed in order to establish the phenotypic effects of mtDNA mutations and the possible role of some common polymorphic variants for LHON penetrance in our subjects. Based on the clinical phenotype, all subjects were categorized as affected (herein, all probands), and unaffected mutation carriers, i.e., individuals carrying a pathogenic mutation, and showing no symptoms of the disease (herein, family members of probands P1 and P2). A thorough full history was taken and ophthalmologic examinations were performed including specialized visual testing, a fundus examination of the optic disc and vascular changes (Table 4), neurological and non-neurological evaluations were also carried out (Table 5).

In the majority of symptomatic cases in our study, LHON presented as painless impairment of the vision in one eye first, then the other eye within an average of several weeks to five months later. In two out of eight probands, visual loss was bilateral at onset (P1 and P4). Our probands were aged between 13–33 years old at onset of the symptoms and time of diagnosis; only one proband (P4, m.11778G>A, H2a haplotype) was identified with disease onset at the age of 55 years. Clinical diagnosis confirmed the severely affected vision in both eyes (VOS and VOD up to 0.5/60, and 0.05/60, respectively) with color vision defects and central scotomas in the visual field. Fundoscopy findings for LHON also showed a disease sequence that includes diffuse pallor of the disc, circumpapillary telangiectatic, tortuous vessels and eventual optic nerve atrophy.

In asymptomatic mutation carriers, examination showed no pathological changes, but the young age of the siblings of proband P1 indicate the need for regular follow-up. 

## 4. Discussion

Leber’s hereditary optic neuropathy (LHON) is the most common inherited mitochondrial disorder. It is a maternally inherited disease and is caused by pathogenic mtDNA point mutations that affect complex I-dependent respiration. This genetic base explains the mitochondrial dysfunction that characterizes LHON disease. The main clinical phenotype shows overt symptoms of blurred vision and gradual painless loss of vision in both eyes [50,51]. The estimated prevalence of LHON in the Serbian population (1 in 526,000) is significantly lower than in Europe (1 in 43,000) [37,52]. Our current study reports the results of Sanger mtDNA sequencing in a group of eight Serbian probands and four unaffected LHON mutation carriers. Our mutational screening revealed two well-known primary LHON mutations, m.11778G>A and m.3460G>A, and one rarely LHON-associated mutation m.8836A>G, which have all been reported previously in the literature [27]. Primary mutation m.11778G>A was detected in two Serbian pedigrees, including two probands and their corresponding four mutation carriers, and in four sporadic cases, but m.3460G>A and m.8836A>G were each detected in singleton probands only. There was a 10:2 homoplasmic to heteroplasmic mutation ratio, whereby heteroplasmy was exhibited in two unaffected m.11778G>A carriers. Moreover, male predominance for LHON was observed in our analysis as the affected male to female ratio was 7:1.

Our finding also revealed the presence of multiple variants that are potentially relevant for LHON pathogenesis and phenotypic expression. A total of five pathogenic secondary mutations are reported in three Serbian subjects harboring primary LHON mutations at nps, m.4216T>C (Y304H) in *ND1*, m.13708G>A (A458T) in *ND5*, m.15257G>A (D171N) and m.15812G>A (V356M) in *CYB-MT* which were all collectively found to be linked and in association with other homoplasmic mtDNA polymorphic changes at nps. Mutations such as m.295C>T, m.489T>C, m.10398A>G, m.11251A>G, m.12612A>G, m.16069C>T, and m.16126T>C are genetic modifiers that affect complex I [53]. Their presence is associated with teenage-years onset of LHON in probands P2 and P7, which suggests their influence on the pathogenic capacity and penetrance of LHON primary mutations and the subsequent, increased risk for affected phenotypes. Also, findings of optic nerve hyperintensity on the MRI of proband P2 contribute to the deliberation of differences in MRI findings according to associated mutations, and early onset of visual loss [54]. In addition, the association of these secondary mutations and other environmental factors, such as tobacco and alcohol could have influenced the penetrance and the early signs and symptoms of LHON in proband P7. In our study, two probands were moderate consumers of cigarettes and/or alcohol (P4 and P7). It is known from previous reports that retinal ganglion cells have selective vulnerability towards complex I mutations, which has a drastic impact on complex I [50,51]. In our study, five out of eight LHON mutations were present in complex I as m.3394T>C, m.3460G>A m.4216T>C, m.11778G>A, m.13708G>A, in *MT-ND1*, *MT-ND4*, and *MT-ND5* subunits. Mutations in mtDNA complex I subunits could be considered as another explanation for how incomplete penetrance in LHON is influenced by genetic background. Further, secondary mutation m.3394T>C in *ND1* (Y30H) has been found to be strongly associated with the m.11778G>A LHON mutation, which contributes to the progression of the disease. According to the literature, the m.3394T>C variant is consistent with the findings in European Caucasian and Chinese LHON patients who harbor m.11778G>A primary mutation, which is a differential expression for haplogroup M9a in Chinese patients, as shown in Figure 2 [55,56], while m.3394T>C rarely co-exists with m.3460G>A LHON cases [57]. 

Based on the predictive software PolyPhen2 in comparison to human amino acid reference sequences identified in UniProt, we re-evaluated all detected primary LHON mutations. Mutations m.3460G>A and m.11778G>A were classified as definitely pathogenic whereas m.8836A>G was possibly damaging on the HumVar model with scores of 1.000, 0.996 and 0.770, respectively. Additional analysis of these mutations by PANTHER (that confirms the generality of the relationship between mutations and the changes in those conversed amino acids), showed that m.3394T>C, m.3460G>A, m.4216T>C, m.11778G>A, and m.15257G>A probably have a damaging effect, however, m.8836A>G was the only one that has a possibly damaging impact; on the other hand, m.13708G>A, and m.15812G>A were recorded as probably having a benign effect. Additionally, m.3394T>C, m.11778G>A, and m.15257G>A were estimated as deleterious on PROVEAN.

So far, there are several studies regarding the genetic profile and diversity of mtDNA in the Serbian population [58,59,60]. Our study determined the association of those specific mitochondrial genetic backgrounds to LHON pathogenic mutations in a group of Serbian subjects; furthermore, we constructed a phylogenetic tree encompassing the studied probands and provided detailed phenotypic characterization of subjects. 

We applied MITOMASTER, which revealed the association of both m.11778G>A and m.3460G>A primary LHON mutations with mtDNA haplogroup R subtypes, with more preferentially to haplogroup U, which is one of the oldest subgroups in Europe and the second most common haplogroup in the Serbian population [61], followed by haplogroup J, and H (50%, 25%, and 16.7%, respectively). These results are in agreement with previous epidemiological studies have been presented the European-specific mtDNA background of LHON primary mutations, for example, a 2007 study by Hudson et al., Herrnstadt et al.’s 2004 review, and the meta-analysis by Newcastle University [62,63,64]. Also, our results are in concordance with other studies from different European geographic regions such as in Russian LHON families, and Italian and Spanish Aragón individuals [65,66,67]. Interestingly, the mutation m.8836A>G in our study is associated with N1b haplogroup, which is present in only 0.39% of Serbian population [68]. Data regarding this mutation in LHON are limited, but in one Saudi-Arabian study, in a single proband it was associated with haplogroup M1a [69], which is common in this population. By phylogeny, M and N macro-haplogroups are close and are both descendants of the haplogroup L3. 

Intriguingly, m.8836A>G substitution is common in the N1b haplogroup branch. It is conceivable that disease-causing mutations with a higher pathogenicity score are less frequent in the population [70]. However we consider the homoplasmic m.8836A>G as a disease relevant mutation supported by (1) the MITOMAP database, which reported that m.8836A>G is a LHON pathogenic variant; (2) our results, which relied on clinical evaluation and phenotypic correlation for a 33 year old male with sequentially poor vision and decreased visual acuity on the left eye first and then on the right eye (VOS 0.5/60 and VOD 3/60), bad vision in the darkness, accompanied by retinal detachment; (3) the bioinformatics analysis for pathogenicity scores found that m.8836A>G is possibly damaging based on by both PolyPhen prediction and PANTHER, which is in contrast to PROVEAN which provided a score of −2.46, that is near the cut-off score of −2.5; (4) high evolutionary conservation in the same haplogroup branch (88.89%); and (5) published papers that provide evidence of pathogenicity [69,71].

Our genetic analysis confirmed that pathogenic mutations in patients clinically suspected of LHON, play a crucial role in the process of differential diagnostics. In proband P4, a cough and cold episode was followed by a simultaneous gradual loss of ocular vision, and post-infectious optic neuropathy was considered. Taking into account other clinical specificities, detection of m.11778G>A, the primary LHON mutation does not favor infectious etiology [72,73]. On the other hand, proband P7 was diagnosed with LHON rather than tobacco-alcohol amblyopia because the LHON mutation m.3460G>A was found accompanied by m.4216T>C, m.13708G>A, m.15275G>A, and m.15812G>A [74]. Interestingly, proband P8 presented with headaches with poor eyesight but without nausea or vomiting, and fundoscopy showed bilateral papilledema retinal detachment, which is occasionally reported in LHON. Detection of mutation m.8836A>G pointed to LHON among other causes of headache associated with bilateral papilledema [75]. Historically, in his original paper, Leber himself reported that some LHON patients presented with concomitant headaches [76,77]. In recent work, Rozen et al. [78] also supposed that some mtDNA LHON mutations could influence the chance of developing a cluster headache. 

## 5. Conclusions

Whole mitochondrial genome analysis in a group of Serbian cases of LHON detected two well-known primary mutations and one less frequent variant, as well as several secondary mutations present in both affected and unaffected carriers. Haplogroup analysis confirmed a European-specific background in the majority of cases, but the less frequent mutation was detected within haplogroup N1b, which is very rare in the Serbian population. Further studies are needed to explore the factors that contribute to the penetrance of LHON mutations and the phenotypic variability of the disease.

## Figures and Tables

**Table 1:** mtDNA mutations and haplogroup characteristics in analyzed subjects.

Subjects	Primary Mutations	Secondary Mutations	Associated Mutations	Haplogroup
P1, mother, sibs	11778G>A	−	−	K1
P2, mother	11778G>A	3394T>C, 4216T>C, 13708G>A	15287T>C, 2755A>G, 3796A>G	J1c
P3	11778G>A	−	−	H1b
P4	11778G>A	−	−	H2a
P5	11778G>A	−	988G>A	U2e
P6	11778G>A	−	−	K1
P7	3460G>A	4216T>C, 13708G>A, 15257G>A,15812G>A	−	J2b
P8	8836A>G	−	−	N1b

The pathogenic primary LHON mutation m.11778G>A was detected in 6 probands and 4 asymptomatic carriers, whereas each m.3460G>A primary LHON mutation and m.8836A>G a mutation were detected in only one proband (P7 and P8, respectively). Both m.11778 and m.3460 are associated with unique consensus sequence of missense mtDNA changes at np.4216 and 13708, further changes at np.15257 and 15812 are associated with m.3460, all are defined in haplogroup J. m.3394T>C showed a preferred association with m.11778 primary LHON pathogenic mutation. Associated mutations for other diseases were detected in association with LHON.

**Table 2:** In silico analysis of the pathogenicity of mtDNA mutations in Serbian LHON probands. Primary mutations are in bold; A.A change: amino acid change.

Variant	Gene	Codon	A.A Change	Status	UniProt ID	Polyphen Prediction	PANTHER	PROVEAN	Previous Reported
m.3394T>C	MT-ND1	30	Y-H	Secondary	P03886	Benign	Probably damaging	Deleterious (−4.40)	Yes
**m.3460G>A**	**MT-ND1**	**52**	**A-T**	**Primary**	**P03886**	**Probably damaging**	**Probably damaging**	**Neutral (−2.36)**	**Yes**
m.4216T>C	MT-ND1	304	Y-H	Secondary	P03886	Benign	Probably damaging	Neutral (3.51)	Yes
**m.8836A>G**	**MT-ATP6**	**104**	**M-V**	**Rare LHON mutation**	**P00846**	**Possibly damaging**	**Possibly damaging**	**Neutral (−2.46)**	**Yes**
**m.11778G>A**	**MT-ND4**	**340**	**R-H**	**Primary**	**P03905**	**Probably damaging**	**Probably damaging**	**Deleterious (−4.74)**	**Yes**
m.13708G>A	MT-ND5	458	A-T	Secondary	P03915	Benign	Probably benign	Neutral (−1.50)	Yes
m.15257G>A	MT-CYB	171	D-N	Secondary	P00156	Benign	Probably damaging	Deleterious (−3.56)	Yes
m.15812G>A	MT-CYB	356	V-M	Secondary	P00156	Benign	Probably benign	Neutral (−0.73)	Yes

Primary mutations are in bold.

**Table 3:** Sub-haplogroups characterization of the detected variants.

Variant	Haplogroup (HG)	Frequency in HG Branch	Conservation
m.3394T>C	J1c	12.60	93.33%
m.3460G>A	J2b	0	91.11%
m.4216T>C	J1c	99.07	24.44%
	J2b	99.14	24.44%
m.8836A>G	N1b	97.97	88.89%
m.11778G>A	J1c	0.77	100.00%
	K1	0	100.00%
	H2a	0	100.00%
	H1b	0.42	100.00%
	U2e	0.60	100.00%
m.13708G>A	J1c	98.76	33.33%
	J2b	98.85	33.33%
m.15257G>A	J2b	99.14	95.56%
m.15812G>A	J2b	98.85	24.44%

**Table 4:** Ophthalmic evaluation of LHON in Serbian subjects.

Subject	Gender	Age at Evaluation	Clinical Picture	Ocular Evaluation	Fundoscopy	Environmental Factors
Mother of P1	Female	45	-	VOS: 1.0-VOD: 1.0.	Yellow optic nerve papilla, tortuous vessels.	-
				PR-VEP: no abnormalities.		
P1	Male	15	Simultaneous binocularly vision loss.	VOS: 2–3/60-VOD: 0.2–0.3/60. Absolute central scotoma. Color vision defects. PR-VEP: bilateral lesion of optic pathways, more right.	Optic disc: clearly demarcated, pale, dilated capillaries peripapillary, blood vessels slightly tortuous flow. Five months later: Optic nerve atrophy.	-
Sister of P1	Female	12	-	VOS: 1.0-VOD: 1.0.	Normal disc appearance.	-
				PR-VEP: high amplitude.		
Bother of P1	Male	4	-	VOS: 1.0-VOD: 1.0.	Normal disc appearance.	-
				PR-VEP: high amplitude.		
Mother of P2	Female	39	-	VOS: 1.0-VOD: 1.0.	Several tortuous capillaries along the optic nerve disc.	-
P2	Male	13	Poor vision of both eyes, right eye then left eye five months later.	VOS: 1.0/60-VOD: 0.1/60. Centrocecal scotoma. Color vision defects.	Optic disc: blurred edges, yellow, peripapillary tortuous dilated capillaries, circumpapillary telangiectatic microangiopathy, swelling of the nerve fiber layer around the disc.	-
				PR-VEP: Bilateral extension of P100 latencies.		
P3	Male	30	Painless gradually low vision in both eyes, first on right eye.	VOS: 3/60-VOD: 4/60.	Pallor of optic nerve papilla.	-
				Central scotoma.		
				PR-VEP: Bilateral extension of P100 latencies on both sides.		
P4	Male	55	Simultaneous vision loss in eyes.	VOS: 2–3/60-VOD: 0.05–0.1/60. Central scotoma.	Left optic disk slightly paler, circumpapillary telangiectasia, and vessel tortuosity.	Alcohol: occasionally consumed;
				PR-VEP: Bilateral extension of P100 latencies on both sides.		Post-infection.
V	Male	20	Sudden loss of vision on left eye then on the right one.	VOS: 4/60-VOD: 0.10/60.	Normal disc appearance.	-
P6	Male	17	Blurred vision, right eye then left eye month later.	VOS: 1.50–2/60-VOD: 0.5–0.75/60. Absolute central scotoma. PR-VEP: bilateral lesion of optic pathways, more right.	Optic nerve atrophy.	-
P7	Female	17	Impaired vision in eyes, left eye then right eye three weeks later.	VOS: 0.5/60-VOD: 0.8–1/60. Bilateral amblyopia, the left non-reactive to light. PR-VEP: decreased amplitude of cortical, prolonged P100 latency only on left eye.	Pale, clear borders, blood vessel tortuosity, numerous striated reflexes in the macula, macular relief disturbed, and no pain when moving the bulbus on left eye.	Smoking: up to ten cigarettes a day. Alcohol: occasionally consumed
P8	Male	33	Sequentially poor vision of eyes within weeks, more pronounced on the left. Bad vision in the darkness. Headache.	VOS 0.5/60-VOD 3/60. Both eyes are non-reactive to light. IOP: 14 mmHg.	Bilateral papilledema. Retinal detachment. Later on optic nerve atrophy.	-

Probands are indicated in bold. IOP: (Intraocular pressure); PR-VEP: (Pattern-reversal Visual Evoked Potentials); VOD (Visus Oculus Dextrus); VOS (Visus Oculus Sinister).

**Table 5:** Neurological and non-neurological evaluation of LHON in Serbian subjects.

Subject	Neurological Evaluation	Non-Neurological Evaluation
Mother of P1	AEP: extension of conduction along intra-axial acoustic pathways	Trivial mitral and aortic regurgitation.
P1	MRI: no abnormalities.	Mild mitral valve prolapse with trivial regurgitation.
	SSEP: giant SEPs above the primary SS cortex of the right hemisphere.	
	AEP: no abnormalities.	
Sister of P1	AEP: extension of conduction along intra-axial acoustic pathways.	-
Brother of P1	AEP: no abnormalities.	-
Mother of P2	MRI: no abnormalities.	QT interval slightly prolonged.
P2	MRI: hyperintense lesion of the right optic nerve	Normal ECG.
P3	MRI: initial cortical reductive changes of the brain supratentorially.	-
	TCD: the optic nerves are thinner in diameter on both sides.	
P4	MRI: chronic microangiopathic changes and periventricular ischemic.	Hypertension.
P5	MRI: increased diameter of the retrobulbar segment of the right optic nerve.	ECG: sinus tachycardia.
P6	MRI: supra-and infratentorial hyper intensive changes and demyelination.	-
	SSEP: asymmetry of latencies of cortical responses to the damage of the left hemisphere.	
	AEP: lower amplitude V wave left.	
P7	MRI: no abnormalities.	Enlarged spleen, aortic effusion.
		Bicuspid aortic valve with mild aortic insufficiency.
		ECG: short PR interval with delta wave.
P8	MRI: no abnormalities.	Gastric ulcer.

Probands are indicated in bold. AEP: (Auditory Evoked Potential); ECG: (Electrocardiogram); MRI: (Magnetic resonance imaging); SS: (Somatosensory); SEPs: (Somatosensory Evoked Responses); SSEP: (Somatosensory Evoked Potential); TCD: (Transcranial Doppler).
